# Patterns and predictors of surgical management in pediatric chronic idiopathic constipation

**DOI:** 10.3389/fped.2026.1955197

**Published:** 2026-10-07

**Authors:** Prisca C. Obidike, Katlyn A. Droke, Grant F. Williams, Cecily A. Wainstein, Trevor C. Jones, Zoë Hemmer, Alison Jung, Bea B. Jeon, Kristy L. Rialon, Lily S. Cheng

**Affiliations:** 1 Division of Pediatric Surgery, Department of Surgery, University of Virginia, Charlottesville, VA, United States; 2 Department of Pediatric Surgery, Texas Children’s Hospital, Houston, TX, United States

**Keywords:** antegrade continence enema, chronic idiopathic constipation, colectomy, colostomy, constipation, ileostomy

## Abstract

**Introduction:**

Chronic idiopathic constipation (CIC) is a common childhood gastrointestinal disorder that substantially affects quality of life and healthcare utilization. Most children are managed successfully with dietary modification, behavioral interventions, and medical therapies. However, many patients experience severe or refractory symptoms that necessitate surgical intervention. We sought to identify patient factors associated with progression to surgical intervention.

**Methods:**

A retrospective multi-institutional cohort study was performed of children (<18 years of age) who presented to a pediatric surgeon for chief complaint of CIC between 2017 and 2022. The primary outcome was major surgical intervention for the management of CIC. Patient characteristics, including demographic characteristics and clinical variables, were queried, and multivariable logistic regression was used to assess the association between patient factors and surgical intervention.

**Results:**

A total of 188 patients were included. Forty patients (21.3%) had a major operation for CIC, including antegrade continence enema (ACE) procedures, intestinal resection, and intestinal diversion. Patients who underwent surgery were older at the time of first visit (mean 9.12 vs. 4.64 years; *p* < 0.001). In multivariable logistic regression, older age at first pediatric surgery visit was independently associated with surgical intervention. Each additional year of age was associated with approximately 30% higher adjusted odds of surgical intervention (adjusted OR 1.29, 95% CI 1.14–1.48; *p* < 0.001). Sex, race, ethnicity, insurance type, and neurodiversity were not significantly associated with surgery.

**Conclusion:**

Approximately one in five patients referred to a pediatric surgeon for refractory constipation undergo surgical intervention to treat constipation. Older age at presentation was associated with surgical treatment.

## Introduction

Chronic idiopathic constipation (CIC) is a common disorder with a significant impact on pediatric populations. CIC is defined as a long-term functional gastrointestinal disorder characterized by persistent symptoms of difficult, infrequent, or incomplete bowel movements without evidence of any identifiable cause. CIC affects a large proportion of children: a pooled global study revealed a prevalence of 12% for children, accounting for approximately 3% of all pediatric primary care visits and up to 25% of pediatric gastroenterology referrals (1, 2). In 2009, it was estimated that children with constipation incurred healthcare costs of $3,430/year compared to the $1,099/year for children without constipation, amounting to an estimated additional $3.9 billion/year in the United States alone (3). The consequences of constipation are not just monetary; they include lifelong physical, mental, and emotional effects. These include fecal impaction, bowel obstruction, megacolon, hemorrhoids, anal fissures, rectal prolapse, stercoral ulceration, and an increased risk of depression (4). Children and adolescents also experience higher rates of social withdrawal, anxiety, and inattention (5). Ultimately, 25% of children with constipation continue to have constipation symptoms well into adulthood (6), resulting in lifelong impacts for many.

Initial management of CIC typically includes a combination of dietary modifications, behavioral interventions, pelvic floor therapy, laxative medications, suppositories, and rectal enemas. However, a significant subset of patients experience refractory symptoms despite lifestyle, dietary, or medical therapy (7). The Neurogastroenterology and Motility Committee of the North American Society for Pediatric Gastroenterology, Hepatology & Nutrition (NASPGHAN) defines pediatric refractory constipation as “ongoing symptoms in children meeting Rome IV criteria who have failed to improve after a minimum of 3 months of age-appropriate conventional therapies with impaired quality of life” and recommends consideration of surgical referral at this point (8, 9). Surgical interventions to treat constipation include antegrade continence enema (ACE) procedures, colonic resections, and intestinal diversion (10). However, there is limited data on the proportion of patients who receive surgical treatment for CIC, the frequency of the various types of surgical intervention, and factors associated with surgical intervention.

The primary objective of this study was to quantify the surgical burden of CIC in children seen by a pediatric surgeon. The secondary aim was to identify patient factors associated with surgical intervention.

## Methods

### Study population

We performed a retrospective cohort study of patients (<18 years old) who presented to a pediatric surgeon with a chief complaint of CIC (International Classification of Diseases, 10th Revision code: K59.04) between March 1, 2017 and August 31, 2022 from two academic tertiary institutions in the United States.

Diagnosis was verified by chart review using the NASPGHAN criteria for chronic refractory constipation and the Rome IV criteria for functional constipation in children (9, 11). Patients with an identifiable cause of constipation were excluded. Exclusion criteria included Hirschsprung disease, anorectal malformations, spina bifida, congenital colorectal disease, inflammatory bowel disease, and other known causes of constipation. The study was approved by the institutional review boards at the University of Virginia and Texas Children's Hospital.

### Variables and data collection

Patient demographic and clinical variables included age at first pediatric surgery visit, sex, ethnicity, race, insurance type, and neurodiversity, defined as documented developmental delay and/or autism spectrum disorder. Results of constipation-related diagnostic testing, including contrast enema (CE), anorectal manometry (AMAN), and colonic manometry (CMAN) were also noted. Abnormal CE results were defined as colonic redundancy or dilatation. Abnormal AMAN results included abnormal resting pressures, relaxation, or pelvic floor dyssynergia. Abnormal CMAN results included decreased high amplitude propagating contractions or diminished contractility in response to stimulant laxatives.

### Outcomes

The primary outcome was receipt of any major surgical intervention for CIC. Surgical procedures included ACE procedures (appendicostomy or cecostomy), colonic resection (total or partial colectomy), intestinal diversion (ileostomy or colostomy), or a combination of these procedures. Minor or diagnostic procedures such as anorectal exams under anesthesia, rectal biopsy, fecal disimpaction, or chemodenervation of the internal anal sphincter were not included.

### Statistical analysis

Continuous variables were summarized using mean and standard deviation or median and interquartile range. Categorical variables were reported as frequencies and percentages. Characteristics were compared between surgical and nonsurgical patients using Student's *t* test or Wilcoxon rank-sum test for continuous variables and Chi-square or Fisher's exact tests for categorical variables.

Multivariable logistic regression was used to evaluate patient factors independently associated with surgical intervention. Given the limited number of surgical events, the multivariable model was kept parsimonious and included age at first surgical visit, sex, ethnicity, and neurodiversity as clinically selected covariates. Race was not included because several categories contained small numbers of patients, which would yield unstable estimates. Insurance type was similarly excluded from the final model because of sparse categories. Adjusted odds ratios with 95% confidence intervals and p-values were reported.

Because patients were included from two institutions, surgical intervention rates were also compared between centers. Study institution was subsequently added to the multivariable model in a sensitivity analysis to evaluate whether potential differences in institutional practice influenced the primary associations. Statistical significance was defined as a two-sided *p*-value <0.05. Analyses were performed using R version 4.4.1 and GraphPad Prism version 11.0.2.

## Results

A total of 188 patients met the study criteria (Supplementary Figure S1). Overall, 105 (55.9%) of patients were male, 32 (17.2%) were Hispanic, and 75 (39.9%) had a documented neurodiverse disorder (Table 1). Median age at first pediatric surgery visit was 4.75 years (IQR 1.95–8.61).

**Table 1:** Characteristics of pediatric patients with chronic idiopathic constipation by surgical status.

Characteristic	Overall	No surgery	Surgery	*p*-value
	(*n* = 188)	(*n* = 148)	(*n* = 40)	
Age at first surgical visit (years), mean (SD)^a^	5.58 (4.37)	4.64 (4.08)	9.12 (3.58)	<0.001
Male sex, *n* (%)	105 (55.9)	83 (56.1)	22 (55.0)	1.000
Hispanic ethnicity, *n* (%)	32 (17.2)	27 (18.5)	5 (12.5)	0.514
Race, *n* (%)				0.429
White	148 (79.6)	113 (77.4)	35 (87.5)	
Black	27 (14.5)	24 (16.4)	3 (7.5)	
Asian	3 (1.6)	2 (1.4)	1 (2.5)	
Other	8 (4.3)	7 (4.8)	1 (2.5)	
Insurance type, *n* (%)				0.445
None	12 (6.4)	11 (7.4)	1 (2.5)	
Private	71 (37.8)	57 (38.5)	14 (35.0)	
Public	100 (53.2)	77 (52.0)	23 (57.5)	
Military	4 (2.1)	2 (1.4)	2 (5.0)	
Other	1 (0.5)	1 (0.7)	0 (0.0)	
Neurodivergent, *n* (%)	75 (39.9)	54 (36.5)	21 (52.5)	0.098
Diagnostic tests				
CE performed, *n* (%)	91 (48.4)	65 (43.9)	26 (65.0)	0.021
Abnormal CE, *n* (%)	28 (30.8)	14 (21.5)	14 (53.8)	0.005
AMAN performed, *n* (%)	48 (25.6)	29 (19.6)	19 (47.5)	<0.001
AMAN abnormal, *n* (%)	21 (43.8)	10 (34.5)	11 (57.9)	0.143
CMAN performed, *n* (%)	16 (8.5)	2 (1.4)	14 (35.0)	<0.001
CMAN abnormal, *n* (%)	13 (81.3)	0 (0)	13 (92.9)	0.025

CE, contrast enema; AMAN, anorectal manometry; CMAN, colonic manometry.

a Calculated from available data (*n* = 104).

### Surgical interventions and timing

Forty patients (21.3%) underwent surgical intervention, while 148 (78.7%) were managed without surgery. Among the 40 surgical patients, 26 (65%) had an ACE procedure alone, three (7.5%) had intestinal diversion alone, four (10%) had colonic resection alone, and seven (17.5%) had more than one procedure. Of the patients who underwent more than one procedure, six (15%) had a combination of resection and ACE while one (2.5%) had ACE followed by ileostomy (Figure 1).

**Figure 1:**
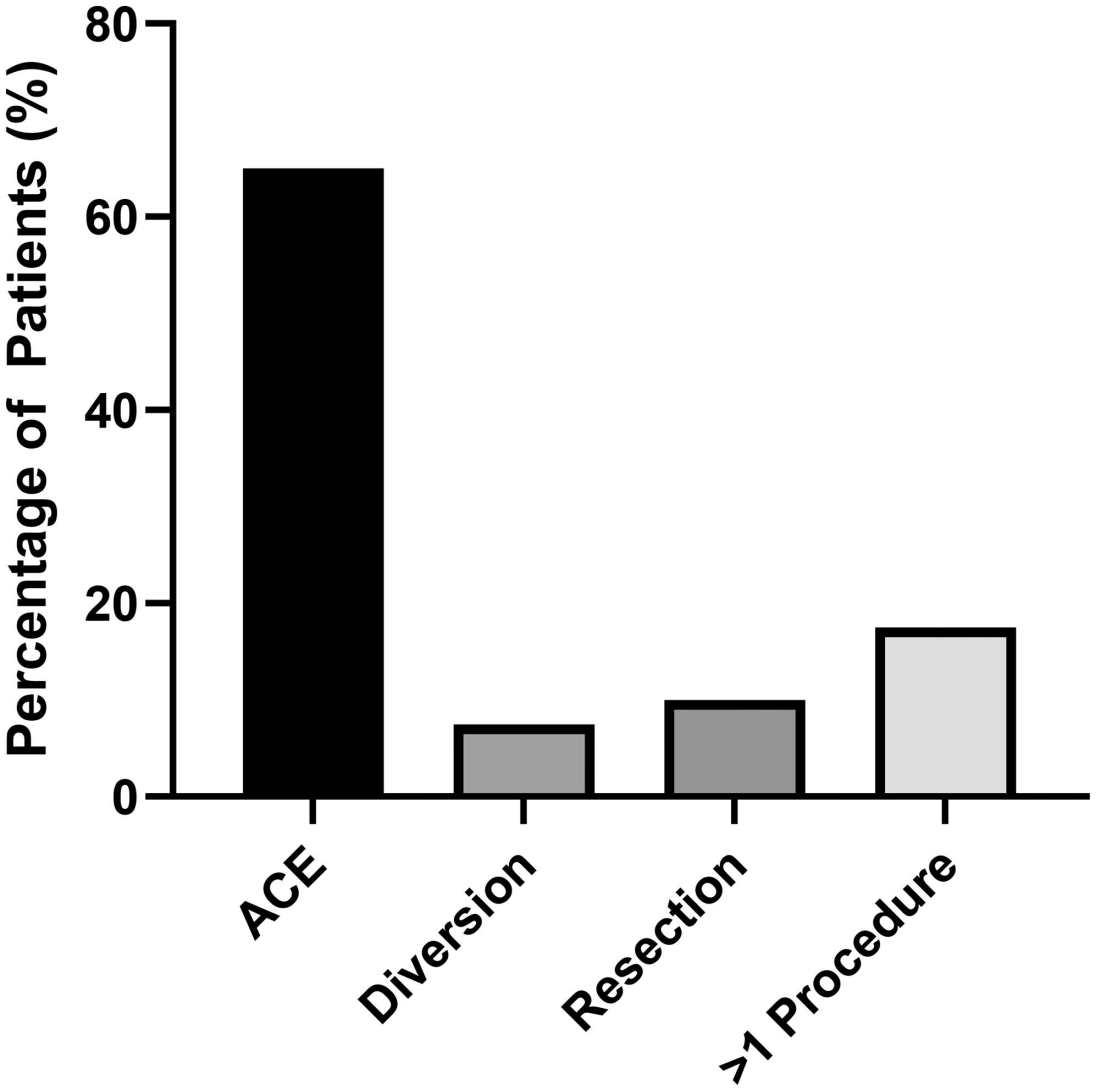
Surgical procedures for chronic idiopathic constipation. Among 188 patients evaluated for chronic idiopathic constipation, 40 (21.3%) underwent a major surgical intervention to treat constipation. Antegrade continence enema (ACE) procedures were the most common operation (65%). Colonic resection (10%) and intestinal diversion (7.5%) were less common, as were patients who had more than one procedure (17.5%).

The date of the patient's first surgical visit for constipation was identifiable for 104 patients. Most patients who had surgery for constipation had an operation within one year of their initial surgical consultation (Supplementary Figure S2). The median time from initial visit to surgery was 295 days (IQR: 149–933).

### Comparison of surgical and nonsurgical patients

Among patients for whom the date of initial surgical consultation was available, patients who underwent surgery were on average older at their first pediatric surgery visit than those who did not undergo surgery (mean 9.12 vs. 4.64 years; *p* < 0.001). There were no significant differences between the surgical and nonsurgical groups in terms of sex, ethnicity, race, or insurance type (Table 1). Neurodiverse disorders were more common among surgical patients than among nonsurgical patients (52.5% vs. 36.5%), although this difference did not reach statistical significance (*p* = 0.098).

Surgical patients were more likely than nonsurgical patients to undergo any diagnostic testing, including CE (65.0% vs. 43.9%; *p* = 0.021), AMAN (47.5% vs. 19.6%; *p* < 0.001), and CMAN (35.0% vs. 1.4%; *p* < 0.001). Among patients who underwent diagnostic testing, surgical patients were more likely to have an abnormal CE (53.8% vs. 21.5%; *p* = 0.005) and abnormal CMAN (92.9% vs. 0%; *p* = 0.025), whereas the proportion with abnormal AMAN did not differ significantly between groups (57.9% vs. 34.5%; *p* = 0.143).

### Factors associated with surgical intervention

In multivariable logistic regression, older age at first pediatric surgery visit was independently associated with surgical intervention (aOR 1.29, 95% CI 1.13–1.47; *p* < 0.001), whereas sex, ethnicity, and neurodiversity were not independently associated with surgery (Table 2). Surgical intervention occurred in 26 of 95 patients (27.4%) at one institution and 14 of 93 patients (15.1%) at the other (*p* = 0.06). In a sensitivity analysis that additionally adjusted for study institution, the association between age and surgical intervention remained similar (aOR 1.27, 95% CI 1.07–1.49).

**Table 2:** Factors associated with surgical intervention for chronic idiopathic constipation in multivariable logistic regression.

Patient characteristics	Adjusted OR (95% CI)	*p*-value
Age at first pediatric surgery visit per year^a^	1.29 (1.13–1.47)	<0.001
Male sex Reference: Female sex	1.10 (0.38–3.21)	0.861
Hispanic ethnicity Reference: Non-Hispanic ethnicity	0.26 (0.03–2.71)	0.262
Neurodiversity present Reference: Neurodiversity absent	1.13 (0.38–3.40)	0.821

a Calculated from available data (*n* = 104).

## Discussion

In this multi-institutional cohort of children with CIC referred to a surgical practice, about one in five underwent operative intervention to treat constipation. An ACE procedure was the most common operation. Older age at initial visit was independently associated with need for surgery, with each additional year corresponding to approximately 30% higher adjusted odds of operative intervention. In contrast, sex, race, ethnicity, insurance type, and neurodiversity were not significantly associated with surgical intervention.

The association of age with need for surgery may represent advanced or prolonged refractory disease with a cumulative failure of medical and behavior therapies, culminating in surgical intervention as a “last resort.” However, it may also suggest a more severe phenotype in older children. Future studies may examine age at symptom onset to differentiate between these two possibilities and determine whether earlier referral or treatment might prevent subsequent surgery.

Diagnostic testing, including motility testing, was also more common among patients who underwent surgery. This association likely reflects, at least in part, confounding by indication. Colonic manometry, in particular, is generally reserved for patients in whom surgery is being contemplated and is used to characterize colonic motor function and guide surgical decision-making (12). Thus, the performance of diagnostic testing may identify a stage in the treatment pathway rather than independently increase the likelihood of surgery. This is reinforced by the finding that the procurement of diagnostic testing, not just abnormal results, was associated with need for surgery.

While this is the first multi-institutional study with the primary objective of quantifying the surgical burden of CIC, other studies have reported similar findings. A multicenter study on fecal incontinence from the Pediatric Colorectal and Pelvic Learning Consortium (PCPLC) found that approximately 30% of children with CIC had an ACE operation with a median age of 9.2 years at operation (13). Notably, they also found that Black patients were significantly less likely to undergo an ACE operation, whereas we noted no significant difference in race between patients who underwent surgery and patients who did not.

Though not statistically significant, neurodiverse disorders were more common in children who underwent surgery. Constipation is common in children with neurodiverse disorders and bowel management can be especially challenging in this population (11). A large portion of neurodiverse patients fail laxative therapy and the PCPLC encourages early consideration of antegrade enemas in this group (14). Larger studies are needed to determine whether neurodiversity independently predicts failure of medical therapy or whether differences in management are motivated by treatment tolerance in this special population.

This study has several limitations. First, the number of patients undergoing surgery was relatively small, limiting statistical power and the number of factors that could be assessed reliably in multivariable analysis. In addition, the duration of individual follow-up was not systematically abstracted. Therefore, patients presenting earlier within the study period may have had greater opportunity to undergo surgery than those presenting later, and the binary surgical outcome does not account for this variation in observation time. Age at initial visit was indeterminate for a substantial proportion of patients. Furthermore, age at the first surgical visit may not accurately represent age at symptom onset, duration of treatment, or timing of referral. NASPGHAN recommends consideration of surgical management for patients with greater than 3 months of refractory symptoms; real world referral patterns and duration of symptoms prior to surgical referral may be ascertained in future studies. In addition, symptom severity, medication adherence, participation in formal bowel management programs, family preferences, and other important clinical factors were not queried in this study. Finally, this cohort was limited to children referred to pediatric surgeons at two tertiary referral institutions, including one institution with a specialized multidisciplinary motility clinic. Accordingly, the observed rate of surgical intervention reflects the operative burden among children referred for surgical evaluation and should not be interpreted as the rate of surgery among all children with CIC. Referral patterns, thresholds for surgical consultation, and institutional diagnostic and treatment algorithms may substantially influence this estimate. Our findings therefore may not be generalizable to the broader population of children with CIC or to centers with different referral and management practices.

Despite these limitations, this study quantifies the operative burden of CIC among children referred to pediatric surgeons at tertiary centers and identifies older age at surgical presentation as an independent marker associated with operative treatment. The need for a major operation in more than 20% of this selected population underscores the clinical burden of refractory constipation and emphasizes a need for more effective medical treatments.

## Conclusion

In this retrospective multicenter study, one in five children referred to pediatric surgery for CIC underwent major surgical intervention to treat constipation. Older age at first surgical visit was associated with an increased likelihood of undergoing surgical intervention.

## Data Availability

The raw data supporting the conclusions of this article will be made available by the authors, without undue reservation.

## Supplementary material

Supplementary Figure S1 Flow diagram of included patients. Identification and selection of the 188 children (<18 years) with chronic idiopathic constipation evaluated by pediatric surgery at two institutions (March 2017–August 2022), after excluding patients with identifiable organic causes of constipation.

Supplementary Figure S2 Time from surgical consultation to operation for pediatric patients with chronic idiopathic constipation. The median time from initial surgical consultation to major operation was 295 days (IQR: 149–933) for the 104 pediatric patients for whom date of initial surgical consultation was available. Most patients who received surgery for constipation had an operation within one year of initial visit and the likelihood of having surgery decreased over time.

## References

[1] Youn JW Joo H Kim S Oh J Yoo H Ko JS. Global prevalence of functional constipation across all age groups according to Rome II–IV criteria, 1999–2025: a systematic review and meta-analysis. Clin Transl Gastroenterol. (2026) 17(6):e01029. 10.14309/ctg.0000000000001029 41919745 PMC13641667

[2] Benninga MA Voskuijl WP Taminiau JAJM. Childhood constipation: is there new light in the tunnel? J Pediatr Gastroenterol Nutr. (2004) 39(5):448–64. 10.1097/00005176-200411000-00002 15572881

[3] Liem O Harman J Benninga M Kelleher K Mousa H Di Lorenzo C. Health utilization and cost impact of childhood constipation in the United States. J Pediatr. (2009) 154(2):258–62. 10.1016/j.jpeds.2008.07.060 18822430

[4] Ren ZY Guo ZY Diao Q Hashimoto K Yang JJ Zhang GF. Constipation is associated with an increased risk of depression: a systematic review and meta-analysis of observational studies. Transl Psychiatry. (2026) 16:395. 10.1038/s41398-026-04138-8 42230550 PMC13444242

[5] Rajindrajith S Ranathunga N Jayawickrama N Van Dijk M Benninga MA Devanarayana NM. Behavioral and emotional problems in adolescents with constipation and their association with quality of life. PLoS One. (2020) 15(10):e0239092. 10.1371/journal.pone.0239092 33044960 PMC7549826

[6] Bongers MEJ Van Wijk MP Reitsma JB Benninga MA. Long-term prognosis for childhood constipation: clinical outcomes in adulthood. Pediatrics. (2010) 126(1):e156–62. 10.1542/peds.2009-1009 20530072

[7] Staller K Neshatian L Lembo A Bharucha AE. AGA clinical practice update on evaluation and management of refractory constipation: expert review. Clin Gastroenterol Hepatol. (2026) 24(2):296–305. 10.1016/j.cgh.2025.09.031 41504675 PMC12788801

[8] Tran DL Sintusek P. Functional constipation in children: what physicians should know. World J Gastroenterol. (2023) 29(8):1261–88. 10.3748/wjg.v29.i8.1261 36925458 PMC10011959

[9] Kilgore AL Rogers Boruta MK Ambartsumyan L Suarez RG Patel D Wood RJ. Evaluation and management of pediatric refractory constipation: recommendations from the NASPGHAN Neurogastroenterology and Motility Committee. J Pediatr Gastroenterol Nutr. (2025) 80(2):353–73. 10.1002/jpn3.12390 39741384

[10] Cheng L Goldstein A. Surgical management of idiopathic constipation in pediatric patients. Clin Colon Rectal Surg. (2018) 31(02):089–98. 10.1055/s-0037-1609023 PMC5825853 29487491

[11] Rasquin A Di Lorenzo C Forbes D Guiraldes E Hyams JS Staiano A. Childhood functional gastrointestinal disorders: child/adolescent. Gastroenterology. (2006) 130(5):1527–37. 10.1053/j.gastro.2005.08.063 16678566 PMC7104693

[12] Dorfman L El-Chammas K Singh A Fei L Mansi S Santucci NR. Exploring diagnostic priorities: the role of colonic manometry in evaluating pediatric patients with intractable idiopathic constipation prior to sacral nerve stimulation. Children. (2024) 11(7):768. 10.3390/children11070768 39062218 PMC11274712

[13] Smith CA Kwon EG Nicassio L Glazer D Avansino J Durham MM. Fecal continence disparities in patients with idiopathic constipation treated at referral institutions for pediatric colorectal surgery. J Pediatr Surg. (2023) 58(1):56–63. 10.1016/j.jpedsurg.2022.09.024 36283846

[14] Short SS Reeder RW Lewis KE Dickie B Grabowski J Sepuha T. The presence of a neurodiverse disorder is associated with increased use of antegrade enema therapy in children with severe constipation: a study from the pediatric colorectal and pelvic learning consortium (PCPLC). J Pediatr Surg. (2022) 57(8):1676–80. 10.1016/j.jpedsurg.2022.04.008 35597676

